# Telegraphing without Wires

**Published:** 1881-04

**Authors:** 


### TELEGRAPHING WITHOUT WIRES.

Prof. Loomis has been for some months experimenting in the West Virginia mountains on his aerial telegraphy, and has succeeded, by running up wires to a certain altitude, in reaching the current of electricity which he claims can be found at that height, and by means of which communication can be had at any distance. It is said the professor has telegraphed to parties eleven miles distant, by merely sending up a kite at each end of the distance to a certain height, attached to which, in place of an ordinary string, was a fine copper wire. When both kites touch the same current, communication was had between them, and messages were sent from one end to the other by means of the ordinary Morse instrument in connection with the instrument invented by Prof. Loomis. He now has a project for a series of experiments from a point on one of the highest peaks on the Alps, in Switzerland, to a similarly situated place in the Rocky Mountains on this side of the world. If this succeeds, of course his invention will rank in importance with that of the electric telegraph itself, and vastly reduce the cost of telegraphing.—*Commercial Bulletin.*

